# Thematic mapping of off-label prescription in psychiatry and its implications for bioethics, human rights, and clinical practice: a scoping review

**DOI:** 10.3389/fpsyt.2026.1705340

**Published:** 2026-02-13

**Authors:** Humberto Müller Martins dos Santos, Luan Felipo Botelho-Souza, Rui Nunes, Ivone Duarte

**Affiliations:** 1 Faculdade de Medicina da Universidade do Porto – FMUP, Porto, Portugal; 2 Centro Universitário Aparício Carvalho – FIMCA, Porto Velho, Rondônia, Brazil

**Keywords:** off-label use, psychiatry, bioethics, human rights, informed consent

## Abstract

**Introduction:**

Mental disorders represent a major global public health challenge, and off-label prescribing in psychiatry has emerged as a common practice given the scarcity of approved therapeutic options. Although often necessary, this practice involves ethical and legal dilemmas that require analysis through the lens of bioethics and human rights. The objective of this scoping review was to map the scientific literature on off-label prescription in psychiatry, identifying its impacts on bioethics, human rights, and clinical practice.

**Methods:**

The study followed PRISMA-ScR guidelines, with searches conducted between July 2024 and Dezember 2025 in PubMed, the Virtual Health Library (BVS), PsycINFO (APA), Web of Science, and the ClinicalTrials.gov register. A total of 1,828 records were identified, of which 38 met the inclusion criteria (last 15 years, with exceptions for foundational historical studies, focusing on psychiatry and bioethical principles). The protocol was prospectively registered in INPLASY (INPLASY202590058). Thematic analysis involved constructing an evidence matrix and keyword co-occurrence networks, using VOSviewer to explore conceptual clusters.

**Results:**

The results highlighted the centrality of the terms off-label, ethics, informed consent, and human rights, demonstrating that the main concerns revolve around clinical responsibility, patient autonomy, and the protection of health as a fundamental right. Special populations, children, adolescents, and pregnant women, stood out as highly vulnerable groups, frequently exposed to prescriptions in contexts of limited evidence. The co-occurrence graph further revealed peripheral subthemes, such as health litigation and pharmaceutical regulation, interconnected with the central core.

**Discussion:**

Thus, the literature reflects a multidimensional concern, predominantly emphasizing ethical and legal dimensions, while pointing to critical gaps in robust clinical evidence and regulatory integration. These findings reinforce the need for clear guidelines, inclusive clinical research, and public policies that ensure safe, equitable practices aligned with bioethical principles and human rights.

**Systematic review registration:**

https://doi.org/10.37766/inplasy2025.9.0058, identifier INPLASY202590058.

## 1 Introduction

Mental disorders represent a significant challenge to global public health. According to the World Health Organization (WHO), approximately one in eight people worldwide lives with a mental disorder, with depression, anxiety disorders, and substance use disorders being the most prevalent (1).

In psychiatry, clinical practice often requires complex therapeutic decisions, particularly given the scarcity of approved treatments for certain clinical conditions, age groups, or vulnerable populations. In such circumstances, off-label prescribing is commonly adopted; this involves the use of medications outside the indications officially approved by regulatory agencies, whether regarding indication, dosage, route of administration, or age group (2).

Although legally permissible and, in many cases, supported by emerging scientific evidence, off-label practices may generate uncertainties regarding treatment efficacy and safety, raising important ethical and legal dilemmas. In this context, the fundamental bioethical principles of autonomy, beneficence, nonmaleficence, and justice are essential for guiding responsible clinical conduct (3). Moreover, access to safe, effective, and high-quality care constitutes a fundamental human right, as established by various international declarations and WHO guidelines (4, 5). Therefore, the decision to prescribe off-label medications requires careful consideration of available scientific evidence and bioethical principles, alongside a commitment to human rights, particularly respect for patient dignity, safety, and well-being.

In this context, the application of thematic mapping through co-occurrence analysis in reviews constitutes a methodological approach for examining the conceptual landscape and the relationships among emerging themes in the scientific literature on off-label prescribing in psychiatry (6). Theoretically grounded in bibliometric analysis and conceptual network theories, this technique allows for the quantitative and qualitative identification of the frequency and proximity with which specific terms and concepts are jointly mentioned in selected publications (7). By revealing semantic connections among central topics such as bioethics, human rights, and implications for clinical practice, co-occurrence analysis fosters an integrated and in-depth understanding of the ethical, legal, and care-related dimensions involved in off-label prescribing (8). Additionally, this analytical strategy strengthens the methodological rigor of scoping reviews by mitigating interpretive biases through the systematic and visual identification of conceptual relationships, highlighting predominant thematic areas, underexplored topics, and critical knowledge gaps (9). Thus, using this approach not only reinforces the scientific and methodological rigor of reviews but also facilitates essential theoretical and empirical advances in understanding psychiatric practice and its bioethical and social implications.

This scoping review aims to map and synthesize the scientific literature on off-label prescribing in psychiatry, with a specific focus on how ethical principles, human rights considerations, and implications for clinical practice are addressed across different psychiatric contexts and populations.

## 2 Methodology

This study adopted a scoping review approach, as recommended by PRISMA-ScR (10), with searches conducted between July 2024 and December 2025 in the electronic databases PubMed, the Virtual Health Library (BVS), and Web of Science. Controlled vocabulary (MeSH terms) and free-text terms were used, including Off-Label Use and Psychiatry, combined with descriptors related to ethics, human rights, informed consent, and legal aspects. Complementary searches were conducted in PsycINFO (APA) and ClinicalTrials.gov. Although records were retrieved, no eligible studies addressing off-label prescribing in psychiatry as a primary focus were identified. Full search details are provided in **Supplementary Material S1**.

As part of the bibliographic search process, the SciSpace platform (https://scispace.com) was additionally employed as a complementary tool for mapping and discovering scientific literature. The use of this technology aimed to broaden the coverage and sensitivity of the search, particularly for the following *a priori* defined thematic descriptors: Off-label prescription and human rights; Informed consent in psychiatric practice; Legal risks and health litigation; Off-label practices in psychiatry in pregnant women; and Off-label practices in psychiatry in the pediatric population. SciSpace was employed exclusively as a mechanism for locating articles and analyzing titles/abstracts, without any automatic content generation, analytical interpretation, or replacement of manual screening by the researchers. All identified sources were critically evaluated by human reviewers, respecting the inclusion, exclusion, and data extraction criteria established in the protocol.

This scoping review protocol was prospectively registered in the International Platform of Registered Systematic Review and Meta-Analysis Protocols (INPLASY) under the registration INPLASY ID: INPLASY202590058 (https://doi.org/10.37766/inplasy2025.9.0058), in accordance with PRISMA-ScR recommendations.

### 2.1 Study selection

The screening process began with the review of titles and abstracts to exclude clearly irrelevant studies. Subsequently, full-text articles were assessed to determine eligibility based on specific inclusion and exclusion criteria. The inclusion criteria include studies published within the last 15 years, with exceptions for historically significant or foundational literature that specifically addresses off-label prescribing practices in psychiatry. The exclusion criteria include articles that did not specifically address psychiatry or that failed to discuss bioethical principles in off-label prescribing.

For reference management, Mendeley Reference Manager (11) was used to organize references, facilitate collaborative review, manage duplicates, and allow detailed annotations during the selection process. The research question and eligibility criteria were defined using the population–concept–context (P: individuals receiving psychiatric care, including adults, children, adolescents, and pregnant women, C: off-label prescription of medications and its ethical, legal, and human rights implications, and C: clinical psychiatric practice, public mental health, and regulatory or policy-related settings) framework, as recommended for scoping reviews.

### 2.2 Selection and eligibility

To include grey literature in this scoping review on the incorporation of bioethical principles in off-label prescribing in psychiatry, a meticulous search was conducted using platforms such as Google Scholar and websites of relevant organizations, including the WHO and national and international health regulatory agencies. MeSH terms such as off-label use and psychiatry were employed. Rigorous inclusion criteria ensured relevance and authority, excluding materials without verifiable authorship or outside the psychiatric or bioethical scope. Selection was carried out through an initial screening of titles and abstracts, followed by detailed evaluation of full texts, with Mendeley (11) used to efficiently organize and review the selected literature. This process ensured the integration of valuable and diverse insights, contributing significantly to a comprehensive understanding of practices and ethical principles involved. Title and abstract screening, full-text assessment, and data extraction were performed independently by two reviewers. Any disagreements were resolved through discussion and consensus, and, when necessary, a third reviewer was consulted to ensure methodological consistency and accuracy.

### 2.3 Organization, data charting, and keyword co-occurrence analysis

A standardized data charting form was developed *a priori* to systematically extract relevant information from each included study. Extracted variables included authorship, year of publication, country or region, type of publication, study objectives, methodological approach, population characteristics, clinical context, primary ethical or legal focus, and key findings related to off-label prescribing in psychiatry.

Data charting was conducted independently by two reviewers. Each study was reviewed in full, and discrepancies in extracted information or thematic attribution were resolved through discussion and consensus. When necessary, a third reviewer was consulted to ensure consistency and analytical rigor.

Thematic synthesis followed a hybrid inductive–deductive approach. Deductive categories were informed by established bioethical principles (autonomy, beneficence, nonmaleficence, and justice) and human rights frameworks, while inductive themes emerged from recurrent patterns identified during the charting process. For instance, studies addressing disclosure practices, shared decision-making, and patient understanding were grouped under the theme *informed consent*, whereas studies focusing on pediatric, adolescent, or pregnant populations contributed to the theme *vulnerable populations*.

To explore the conceptual structure and thematic relationships within the literature, a keyword co-occurrence network analysis was performed using VOSviewer software. “Terms” were defined as author-provided keywords and standardized descriptors extracted from titles and abstracts of the included studies. Prior to analysis, keywords were reviewed and normalized through manual inspection to ensure terminological consistency.

Keywords were organized into a binary incidence matrix, in which each row represented a bibliographic reference and each column corresponded to a term. This matrix enabled the identification of both the frequency of individual terms and the co-occurrence of term pairs within the same reference. A co-occurrence matrix was subsequently generated and visualized through heatmaps and network graphs, allowing identification of thematic density, central concepts, and clustering patterns.

Indicators such as degree centrality, connection density, and cluster modularity were considered to describe both robust thematic cores and emerging subnetworks in the literature. It is important to note that keyword co-occurrence reflects the salience and relational prominence of concepts within the literature, rather than the strength, quality, or effectiveness of empirical evidence. Accordingly, the bibliometric maps were interpreted as descriptive representations of thematic emphasis and conceptual organization, supporting the identification of trends, gaps, and future directions in the field.

### 2.4 Ethical considerations

This study adhered to ethical guidelines for reviews, respecting copyright and the confidentiality of information. As the research did not directly involve human participants, ethical concerns related to participation were minimized.

## 3 Results

**Figure 1:**
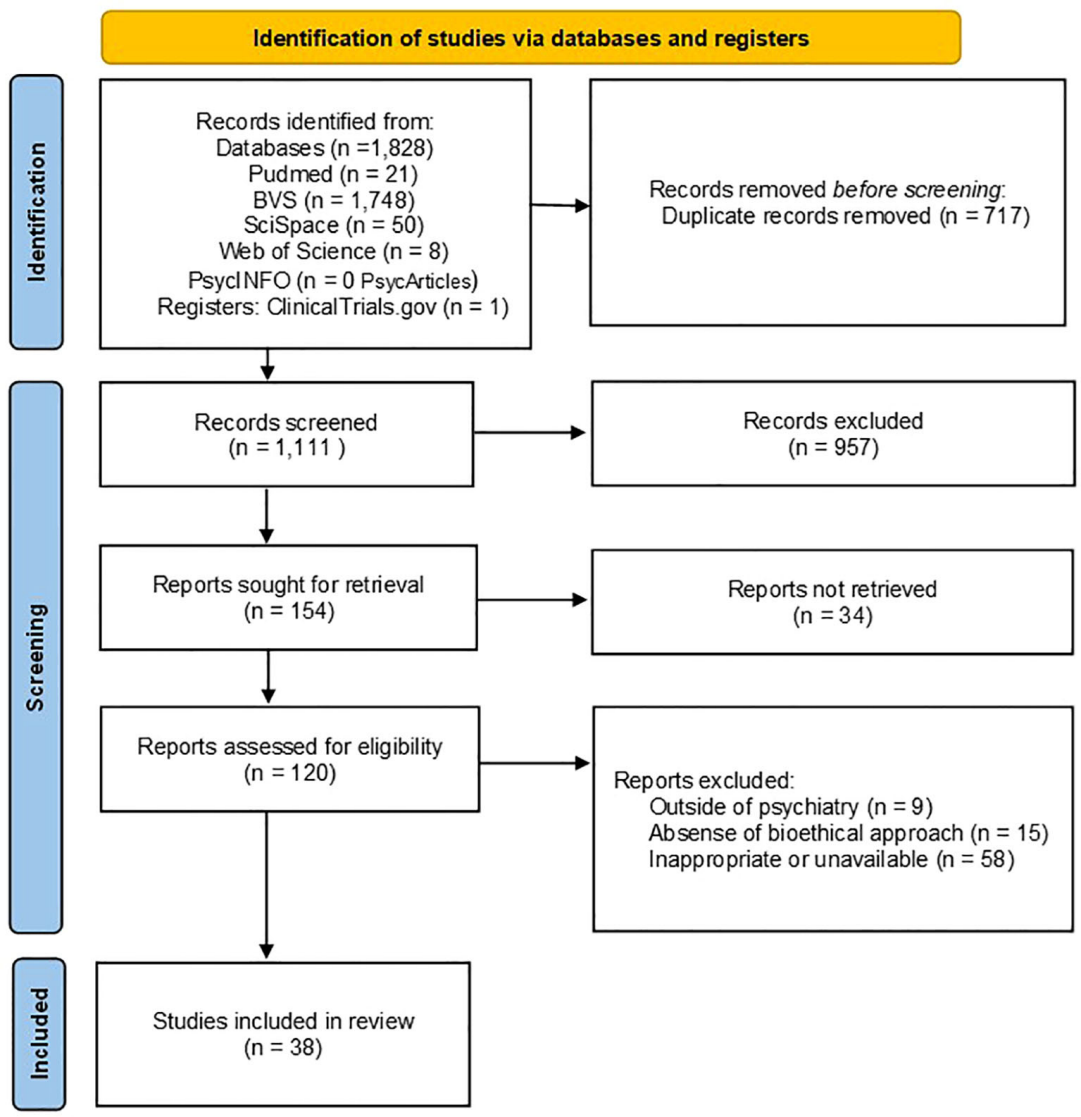
PRISMA-ScR flow diagram of the study selection process.

The study selection process is summarized in the PRISMA-ScR flow diagram (**Figure 1**). A total of 1,828 records were identified through bibliographic database searches, including PubMed (*n* = 21), the Virtual Health Library (BVS; *n* = 1,748), SciSpace (*n* = 50), Web of Science (*n* = 8), and PsycINFO (PsycArticles; *n* = 0). In addition, one record was identified through a trial registry search (ClinicalTrials.gov; *n* = 1). After the removal of 717 duplicate records, 1,111 unique records remained and were screened based on titles and abstracts. Of these, 957 records were excluded for not meeting the eligibility criteria, resulting in 154 reports sought for retrieval. A total of 34 reports could not be retrieved, and 120 full-text reports were assessed for eligibility. Among these, 82 reports were excluded for reasons including studies conducted outside the psychiatric context (*n* = 9), absence of a bioethical or human-rights approach (*n* = 15), and inappropriate or unavailable publication format (*n* = 58). Ultimately, 38 studies met all inclusion criteria and were included in this scoping review. These studies provide a robust foundation for mapping the main ethical, legal, and clinical themes related to off-label prescribing in psychiatry, as well as their implications for human rights and mental healthcare practice.

The Evidence Matrix (**Table 1**) systematizes the main characteristics of the studies selected in this scoping review, encompassing information such as authorship, year, venue, type of publication, methodological approach, and the nature of the literature. This tool provides a comprehensive overview of thematic distribution, methodological diversity, and the primary trends in scientific production related to off-label prescribing in psychiatry. The use of the matrix reinforces the transparency and analytical rigor of the mapping process, enhancing the synthesis of findings and supporting the critical analysis of gaps and convergences identified in the literature.

**Table 1:** Systematized evidence matrix of the scoping review.

Author (year)	Abbreviated title	Publication venue/Journal	Country/Region	Publication type	Literature type	Category	Abstract/Main findings	Themes	Keywords
Blease et al. (2018) (27)	Informed consent in psychotherapy	Journal of Contemporary Psychotherapy	USA/UK	Review	White	Informed consent	Debates the need for informed consent based on evidence and individual factors.	Informed consent; psychotherapy; evidence-based practice	Informed consent, psychotherapy, ethics, disclosure, evidence-based practice
Blondon et al. (2008) (34)	Off-label prescribing	Revue Médicale Suisse	Switzerland	Review	White	Legal risks/Litigation	Analyzes ethical–legal challenges, risks, and practices in off-label prescribing.	Off-label prescribing; regulatory issues; medical ethics	Off-label, psychiatry, regulation, informed consent, medical liability
Braüner et al. (2016) (40)	Off-label prescription in child psychiatry	Journal of Clinical Psychopharmacology	Denmark	Original article	White	Pediatric population	High prevalence of off-label prescribing in children/adolescents; highlights risks and need for monitoring.	Off-label in child psychiatry; polypharmacy; safety	Off-label, child psychiatry, psychotropics, polypharmacy, risks, prevalence
Chisolm and Payne (2016) (51)	Psychotropic drugs in pregnancy	BMJ (online)	UK	Review	White	Pregnant population	Reviews psychotropic drug management during pregnancy; highlights gaps in evidence.	Psychotropics in pregnancy; clinical management; fetal risk	Pregnancy, psychotropics, safety, off-label, clinical management, fetal risk
Clemow et al. (2015) (50)	Medicines in pregnancy forum proceedings	Therapeutic Innovation and Regulatory Science	International	Forum report	White	Pregnant population/Regulation	Highlights the need for innovative regulatory and ethical strategies in research with pregnant women.	Ethics in pregnancy medication; regulation; clinical research	Ethics, pregnancy, medicines, regulation, clinical research
Coleman and Rosoff (2011) (12)	Legal regulation of physicians’ off-label prescribing	Notre Dame Law Review	USA	Original article	White	Human rights/Ethics/Regulation	Argues for legal regulation of off-label prescribing and its impact on patient autonomy.	Legal regulation; off-label prescribing; right to health	Regulation, off-label, legislation, medical liability, right to health
Comanor and Needleman (2016) (19)	Law, economics, and medicine of off-label prescribing	Washington Law Review	USA	Original article	White	Human rights/Economics/Health	Examines economic and legal impacts of off-label prescribing on public health and the pharmaceutical market.	Health economics; off-label; drug policy	Economics, off-label, regulation, drug policy, legal analysis
Coppola et al. (2024) (52)	Medicines in pregnancy: extrapolation framework	Pharmacometrics and Systems Pharmacology	International	Review	White	Pregnant population/Pharmacology	Proposes methods for extrapolating pharmacology data to pregnant women.	Pharmacology in pregnancy; clinical extrapolation; evidence gaps	Pregnancy, clinical pharmacology, extrapolation, knowledge, safety
Diusembayeva et al. (2022) (49)	Clinical trials in pregnant women	Karagandy University – Biology, Medicine…	Kazakhstan	Review	White	Pregnant population/Clinical research	Analyzes obstacles and solutions for conducting research with pregnant women.	Clinical trials in pregnancy; clinical research; regulation	Clinical trials, pregnancy, clinical research, regulation, medicines
Drenska and Getov (2017, 2020) (20, 21)	Approaches for the regulation of off-label use in the EU	Acta Medica Bulgarica/Macedonian Pharmaceutical Bulletin	European Union	Review	White	Human rights/Regulation	Examines different approaches to regulating off-label use in the EU.	European regulation; off-label use; pharmaceutical policy; health policies	Europe, off-label, regulation, public policy, pharmacovigilance, health policy
Gore et al. (2017) (45)	Pediatric off-label and unlicensed drug use	Current Clinical Pharmacology	India	Review	White	Pediatric population	Discusses risks/benefits of pediatric off-label use and the need for monitoring.	Pediatric off-label use; unlicensed use; clinical implications	Pediatrics, off-label, medicines, risks, pharmacovigilance
Jackson et al. (2012) (24)	Off-label prescribing in older patients	Drugs and Aging	International	Review	White	Elderly population	Addresses challenges of off-label prescribing in the elderly, pharmacovigilance, and risk.	Off-label in older adults; geriatric pharmacotherapy; safety	Elderly, off-label, medicines, risks, safety
Lehman and Aroney (2024) (39)	Assessing off-label medication use in psychiatry	Australasian Psychiatry	Australia	Review/Protocol	White	Psychiatry/Clinical practice	Structured protocol for evaluating off-label use in psychiatry.	Psychiatry; off-label assessment; decision-making	Psychiatry, off-label, clinical evaluation, guidelines, medical decision
Matsui (2015) (48)	Ethics of studies of drugs in pregnancy	Pediatric Drugs	Canada	Review	White	Pregnant population/Research ethics	Advocates for the ethical inclusion of pregnant women in clinical research.	Research ethics; medications in pregnancy; justice	Ethics, pregnancy, clinical research, autonomy, justice, risks
McMurray (2002) (29)	Applying principles of informed consent in psychiatry	Canadian Psychiatric Association Bulletin	USA	Review/Commentary	White	Informed consent	Discusses the integration of consent into psychiatric routines and practical barriers.	Informed consent; psychiatry; clinical practice	Informed consent, psychiatry, medical ethics, disclosure, autonomy
de Melo (2024) (23)	Right to health and quality of generic medicines	Themes in Interdisciplinarity	Brazil	Original article	White	Human rights/Quality in health	Analyzes the right to health from the perspective of safe and equitable access.	Right to health; drug quality; public policy	Right to health, medicines, quality, regulation, access
Minghetti et al. (2022) (44)	Off-label in child/adolescent psychiatric emergencies	Pediatric Emergency Care	Italy	Original article	White	Pediatric population/Psychiatric emergencies	Highlights recurrent use and challenges in child/adolescent psychiatric emergencies.	Pediatric psychiatric emergencies; psychopharmacology; epidemiology	Off-label, child psychiatry, emergencies, psychopharmacology, epidemiology
Narayan (2015) (28)	Informed consent in psychiatric practice	Eastern Journal of Psychiatry	India	Review	White	Informed consent	Addresses ethical/legal foundations of consent in psychiatry.	Informed consent; psychiatry; decision-making capacity	Informed consent, psychiatry, ethics, capacity, decision
Nechita et al. (2015) (30)	Informed consent in psychiatric medical care	Rev. Med. Chir. Soc. Med. Nat	Romania	Original article	White	Informed consent	Quantitative study on perception and practice of consent in psychiatry.	Informed consent; medical perception; psychiatry	Informed consent, psychiatry, ethics, medical staff, perception
Neilson and Chaimowitz (2014) (33)	Informed consent to treatment in psychiatry	Canadian Journal of Psychiatry	Canada	Review	White	Informed consent	Highlights the challenges of consent in mental health and proposed solutions.	Consent in psychiatry; decision-making	Consent, psychiatry, decision-making, ethics
Neville et al. (2014) (46)	Off-label use of drugs in children	Pediatrics	USA	Review	White	Pediatric population	Addresses the extent and limitations of off-label use in children.	Off-label in children; safety; clinical practice	Pediatrics, off-label, medicines, safety, clinical practice
Ng (2024) (15)	Informed consent in clinical practice	Journal of the Royal College of Physicians of Edinburgh	UK	Review	White	Informed consent	Describes longstanding and emerging challenges of consent in clinical care.	Informed consent; emerging clinical challenges; ethics	Informed consent, clinical practice, ethics, psychiatry
da Silva Nobre (2013) (32)	Off-label prescription in Brazil and the USA	Ciência & Saúde Coletiva	Brazil/USA	Review	White	Consent/Litigation	Highlights the need for informed consent and legal analysis in Brazil/USA.	Off-label in Brazil and the USA; legislation; insurance coverage	Off-label, Brazil, USA, legislation, informed consent, health insurance
Rabelo Jr. and Goulart (2023) (14)	Off-label prescription: analysis of civil liability	Revista Ibero-Americana de Humanidades, Ciências e Educação	Brazil	Original article	White	Legal risks/Consent	Analyzes physicians’ civil liability through the lens of informed consent.	Medical liability; consent; off-label prescribing	Off-label, civil liability, informed consent, medical ethics
Raposo (2020) (13)	Consumer-patient protection	Advanced Pharmaceutical Bulletin	Portugal	Original article	White	Human rights/Legality	Study on consumer/patient protection and responsibility in off-label prescribing.	Consumer protection; off-label; patient rights	Consumer, off-label, patient rights, legislation, pharmacovigilance
Rusz et al. (2021) (25)	Off-label medication: complex practical aspects	Int. J. Environ. Res. Public Health	International	Review	White	Clinical practice/Public health	Details the practical, legal, and ethical challenges of off-label use.	Off-label: concept and practice; regulatory complexity	Off-label, medicines, regulation, clinical practice, ethics
Santana and Silva (2022) (37)	Health litigation: unapproved medicines	Revista Missioneira	Brazil	Original article	White	Litigation/Public policy	Analyzes health litigation with a focus on unapproved medicines.	Health litigation; unapproved medicines; ANVISA	Litigation, medicines, ANVISA, public health, lawsuits
Sharma et al. (2016) (43)	BAP position statement off-label in children/adolescents	Journal of Psychopharmacology	UK	Guideline	White	Pediatric population/Guideline	BAP statement on off-label prescribing in child/adolescent psychiatry.	Child psychiatry guidelines; off-label prescribing	Child psychiatry, off-label, guidelines, psychotropics, ethics
Spector and Marquez (2011) (35)	Off-label prescribing, PDR, and the court	Journal of the Louisiana State Medical Society	USA	Original article	White	Litigation/Legality	Analyzes the influence of the PDR in judicial rulings on off-label use.	Judicial standards; PDR; medical liability	Off-label, judicial standard, PDR, medical liability, jurisprudence
Syed et al. (2020) (36)	Law and practice of off-label prescribing	J. Am. Acad. Psychiatry and the Law	USA	Review	White	Litigation/Legality	Reviews the legal evolution of off-label prescribing, litigation risks, and medical protection standards.	Practice and legislation; medical promotion; off-label	Off-label, legislation, medical promotion, liability, ethics
Vanderhoof et al. (2023) (26)	Off-label medications for addictive disorders	Current Psychiatry	USA	Review	White	Special population/substance use disorders	Reviews off-label use in addictive disorders.	Off-label in addiction; psychiatry; alternative treatments	Off-label, psychiatry, addiction, psychotropics, alternative treatments
Vesely (2013) (42)	Guideline for off-label use of psychotropic drugs in children	Neuropsychiatrie	Austria	Guideline	White	Pediatric population/Guideline	Guideline for off-label psychotropic use in children/adolescents.	Guidelines for pediatric off-label; ethics	Off-label, psychotropics, guidelines, children, adolescents, ethics
Weld et al. (2021) (47)	Ethical issues in research on pregnant women	British Journal of Clinical Pharmacology	UK	Review	White	Pregnant population/Research ethics	Discusses challenges and solutions for research in pregnant/lactating women.	Research ethics; pregnant women; therapeutic use	Ethics, clinical research, pregnancy, therapeutic use, medicines

**Figure 2:**
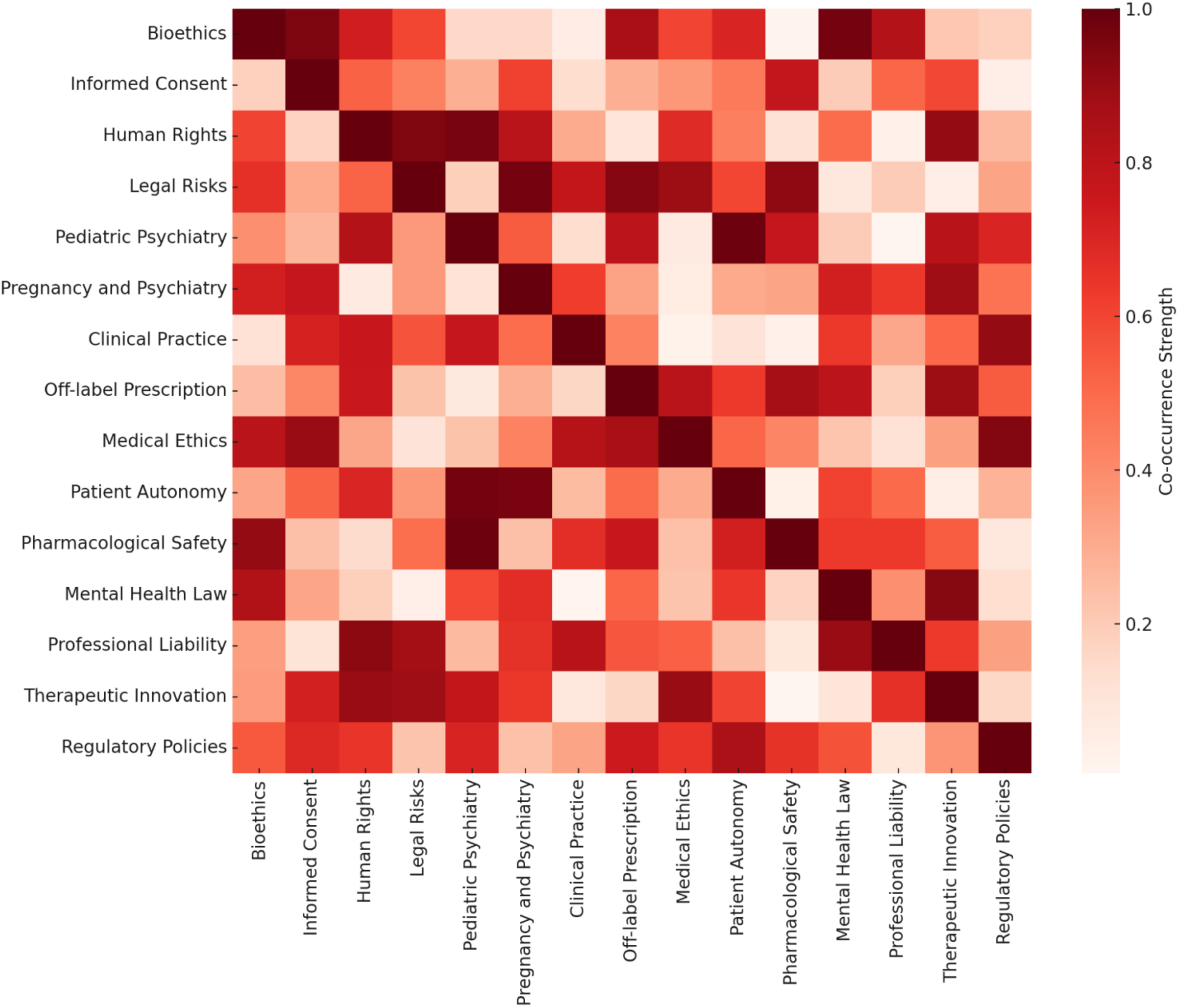
Heatmap of co-occurrences of themes and keywords related to off-label prescribing in psychiatry.

The analysis of the keyword co-occurrence heatmap (**Figure 2**) revealed the presence of robust thematic clusters in the literature on off-label prescribing in psychiatry. High co-occurrence was observed among the terms off-label, ethics, informed consent, and human rights, suggesting that debates on unconventional prescribing practices are primarily concentrated on the ethical and legal dimensions of care. Areas of greater intensity in the map indicate that bioethical and legal concerns are addressed transversally across most publications, highlighting a multidimensional approach to the phenomenon.

Additionally, frequent connections were identified between themes related to special populations, such as pediatrics and pregnancy, which were often associated with terms like safety and vulnerability. These associations reflect the recurring concern in the literature regarding the risks and specific challenges of off-label prescribing in these groups, particularly in the context of scarce robust clinical evidence and the need to ensure additional protection for vulnerable patients.

**Figure 3:**
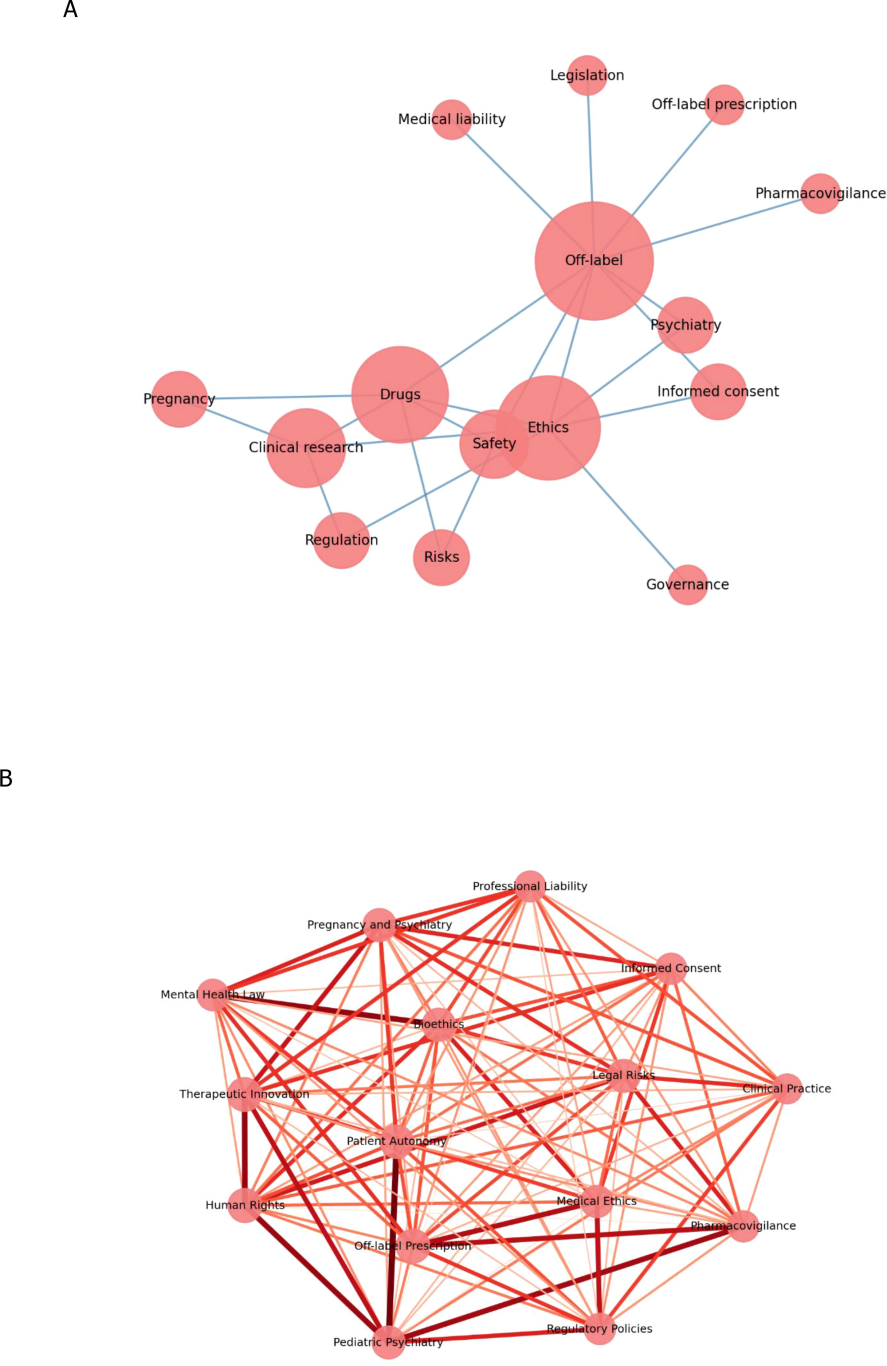
Co-occurrence graph of themes and keywords **(A)** with connection strength **(B)**.

The co-occurrence graph (**Figure 3**) reinforces these findings by visually illustrating the interrelationships among central themes. The core of the graph, composed of off-label, ethics, psychiatry, and informed consent, reveals a highly connected structure, with these terms functioning as articulating axes of discussion and guiding the debate around clinical responsibility and patient autonomy. Peripheral clusters, particularly those related to litigation, vulnerable populations, and regulatory policy, indicate the emergence of specialized subthemes, often interconnected with the central core through concepts such as bioethics and responsibility.

These results point to a body of literature that, while multidisciplinary, maintains a predominant focus on the normative, ethical, and legal dimensions of off-label prescribing in psychiatry. Furthermore, gaps are identified, including the need for greater integration between debates on litigation, clinical guidelines, and safety in special populations. These findings highlight the importance of collaborative and cross-sectoral approaches in the development of more comprehensive recommendations and safer clinical practices.

## 4 Discussion

### 4.1 Relationship between off-label prescribing and human rights

The literature reviewed suggests that off-label prescribing places fundamental human rights principles under tension, particularly the right to quality healthcare, individual autonomy, and protection against unnecessary risks. Although off-label prescribing represents a legitimate and sometimes necessary therapeutic alternative in many clinical contexts, it also raises important ethical, legal, and public health challenges that cannot be overlooked (12, 13).

Across the reviewed studies, informed consent consistently emerges as a *sine qua non* condition for ensuring that off-label use aligns with respect for patient dignity. Physicians have a duty to disclose not only the potential benefits and risks of treatment but also the fact that the proposed use of the medication lies outside the officially approved indications. The omission of this information directly violates the principle of autonomy, which guarantees the patient the right to participate consciously in decisions about their own treatment (14). In psychiatric contexts, where consent may be particularly vulnerable due to patients’ clinical conditions, these requirements become even more pressing (15). This perspective is reinforced by recent work emphasizing the need to align off-label prescribing with human-rights–based frameworks and quality-of-life considerations, particularly in vulnerable psychiatric populations (16, 17).

Empirical evidence further supports these ethical concerns. Luo et al. (18) demonstrate that off-label prescribing in psychiatric settings is frequently driven by gaps in approved indications and clinical urgency, particularly in complex or treatment-resistant cases. Their findings highlight how structural limitations in regulatory approval intersect with clinicians’ ethical responsibilities, reinforcing the need for transparent decision-making and robust informed consent to safeguard patients’ autonomy and right to adequate care (18).

Several studies included in this review highlight that, from a legal perspective, the absence of a specific regulatory framework or clear guidelines on off-label use may result in civil and ethical liability for physicians and institutions. As Raposo (13) emphasizes, harm to patients, unexpected adverse effects, or nonconsented off-label treatments may lead to liability for negligence or malpractice, especially when there is no robust evidence of efficacy or when clear communication with the patient is lacking (13). This concern is further amplified by the lack of uniformity across international regulatory systems, which adopt diverse positions regarding the permissibility and oversight of off-label prescribing (19–21).

Ethical analyses of off-label psychotropic use further underscore these tensions. Smogur et al. (22) highlight that ethical acceptability is often negotiated in contexts of clinical uncertainty, where prescribers balance beneficence against nonmaleficence in the absence of robust evidence. Their findings reinforce that off-label prescribing is not inherently unethical but becomes ethically problematic when transparency, proportionality, and patient involvement are insufficient (22).

The reviewed literature indicates that the right to health—understood as access to safe, effective, and evidence-based care—may be compromised when off-label prescribing occurs without adequate scientific support (23). de Melo (23) argues that patients subjected to treatments whose risk profiles are not fully understood, or whose efficacy has not been validated for the treated condition, may have their right to health violated. This is particularly problematic in vulnerable populations, such as children, the elderly, and individuals with mental disorders, who are often more exposed to off-label prescribing and less protected by legal mechanisms of shared decision-making (19).

From a human-rights and bioethical perspective, we argue that off-label prescribing should not be automatically dismissed, as in many cases it represents the only viable therapeutic option in the absence of approved treatments, particularly in areas neglected by conventional clinical research (24–26). However, this legitimacy does not exempt the need for sustained ethical and scientific rigor. Balancing therapeutic innovation with the imperatives of human rights—particularly the protection of patient autonomy and safety—remains an ongoing challenge that requires careful regulation, continuous ethical training for professionals, and institutional mechanisms of oversight and accountability (12, 27).

### 4.2 Informed consent in psychiatry

Studies included in this review indicate that informed consent is one of the most challenging yet fundamental ethical pillars of contemporary psychiatric practice. Although widely recognized as both a legal and moral requirement, its implementation in mental health continues to present significant gaps, primarily due to clinical complexity and variability in patients’ decision-making capacity (28, 29).

The literature consistently reports that informed consent in psychiatry requires heightened professional attention, given the impact of psychiatric conditions on patients’ understanding, judgment, and voluntariness (28, 30). Disorders such as schizophrenia, manic episodes, or severe depression can temporarily compromise individual autonomy, making adaptive and individualized approaches imperative to ensure that the information provided is fully understood (27).

In this context, effective informed consent practices must go beyond mere documentary formality. As discussed by McMurray (29), it is essential that professionals rely on clear and empathetic communication strategies, emphasizing active listening and comprehension checks throughout the therapeutic process (29). When properly conducted, consent strengthens not only the legality of the medical act but also the therapeutic alliance and the patient’s autonomy, fostering engagement in the therapeutic plan (28).

Consistent with these findings, Rosland et al. (31) emphasize that shared decision-making and patient-centered communication play a central role in ethically justifiable off-label prescribing. Their work indicates that meaningful dialogue and iterative consent processes may mitigate ethical risks, particularly in psychiatric settings, where decisional capacity can fluctuate over time (31).

In Brazil and other international contexts, most professionals acknowledge the ethical importance of informed consent (30, 32). However, challenges remain regarding the standardization and systematization of its application, especially in involuntary hospitalization settings or with patients in crisis. A quantitative study conducted in Romania demonstrated variations in medical staff perception according to age group, suggesting that generational factors may influence the appreciation and management of consent (30).

Furthermore, informed consent in psychiatry should be understood as a dynamic process that must be continuously reassessed. The literature emphasizes that a patient’s mental state may change over time, requiring professionals to adopt a vigilant and sensitive ethical posture to resume the consent process whenever necessary (27, 29).

Another critical aspect concerns consent in research contexts involving psychiatric patients. According to Narayan (28), in situations where decision-making capacity is compromised, it is necessary to seek consent from legal representatives to safeguard participants’ ethical integrity. Borderline practices, such as covert medication, raise ethical controversies. Although sometimes justified as protective measures, such practices demand strict regulation and profound ethical reflection (28).

Studies such as those of Blease et al. (27) highlight the need to update professional codes of ethics, arguing that the informed consent process should incorporate scientific evidence on treatment efficacy and acknowledge nonspecific factors, such as the therapeutic alliance and patient expectations.

Based on this body of evidence, we argue that informed consent in psychiatry should be understood as a continuous, adaptable, and relational process. This requires investment in ethical and communicational training for mental health professionals, as well as the strengthening of institutional policies that promote patients’ autonomy and dignity at all stages of care (33).

### 4.3 Legal risks and health litigation: implications of off-label prescribing

The literature reviewed describes off-label prescribing as entangled in a complex web of legal risks and potential health litigation. The challenge lies in the delicate balance between the clinician’s autonomy and the legal responsibilities arising from their actions, particularly when the therapeutic outcome does not meet expectations or results in patient harm (34, 35).

From a legal standpoint, off-label prescribing occupies an intersection between legality and clinical prudence. In the USA, for example, although the Food and Drug Administration (FDA) allows physicians to prescribe off-label medications, restrictions imposed on the commercial promotion of such uses by pharmaceutical companies have generated legal and ethical controversies, particularly regarding medical freedom of expression and transparency in scientific communication (36). Additionally, US courts frequently use the *Physician’s Desk Reference* (PDR) as a parameter to assess whether medical conduct aligns with recognized standards of care. However, this practice may unduly restrict medical autonomy by conditioning the validity of a clinical decision on whether it is included in official reference materials (35).

In the Brazilian context, although there is no explicit prohibition of off-label prescribing, the practice is subordinated to the requirement of obtaining robust and documented informed consent. According to da Silva Nobre (32), national legislation permits prescribing outside of approved indications, provided that the professional delivers clear, complete, and accessible information to the patient regarding the risks, benefits, and uncertainties involved. Failure to comply with this requirement may result in civil liability for medical negligence, particularly in cases of adverse events or lack of therapeutic efficacy (32).

Health litigation, a growing phenomenon in several public health systems, also directly affects off-label prescribing. Patients who experience therapeutic failure or perceive harm may turn to the judiciary in search of reparation. Although recent case law tends to recognize the complexity of clinical decision-making and offers physicians some degree of protection, the risk of litigation remains a real and constant concern in clinical practice (36). Judicial analyses discussed in the reviewed studies reveal that the absence of adequate documentation, particularly regarding informed consent, is a decisive factor in determining liability and punishment (37).

Empirical evidence suggests that medicolegal pressure and fear of litigation may influence psychiatrists’ prescribing behaviors, contributing to defensive practices such as cautious dosing, increased monitoring, or avoidance of therapeutic innovation, particularly in vulnerable populations (38).

From an ethical and practical perspective, it is essential that the decision to prescribe off-label be grounded in consistent scientific evidence, even if derived from observational studies, case reports, or expert consensus. In fields such as psychiatry or rare diseases, where randomized clinical trials are scarce, this practice may paradoxically represent the only viable therapeutic option. However, the lack of clear regulation regarding the dissemination of such information by pharmaceutical companies remains a sensitive issue, as it may both deprive physicians of useful data and create precedents for inappropriate marketing (36).

Therefore, the practice of off-label prescribing must be accompanied by a cautious, ethically sound, and legally grounded approach (36). Professionals are advised to maintain accurate records of the clinical justifications for nonapproved drug use, ensure clear documentation of informed consent, and stay attentive to regulatory updates (39). Prudence in conduct and transparency in dialogue with patients not only reinforce the clinical legitimacy of off-label prescribing but also minimize the legal risks associated with health litigation (36, 39).

### 4.4 Off-label psychiatric prescribing in special populations

#### 4.4.1 Child and adolescent psychiatry

The use of psychotropic medications outside approved indications is widespread in child and adolescent psychiatry, primarily due to the limited availability of formally approved therapeutic alternatives for this age group. Recent studies indicate that off-label prescribing constitutes a significant portion of clinical practice in this population, presenting considerable ethical, legal, and scientific challenges (40).

The prevalence of off-label prescribing in psychiatric settings for children and adolescents is high. A survey by Braüner et al. (40) found that 32.3% of all outpatient prescriptions were considered off-label, with 41.6% of patients receiving at least one medication outside formal indications.

This concern is reinforced by evidence from Taurines et al. (41), who describe extensive off-label psychotropic use in children and adolescents with neurodevelopmental and psychiatric disorders. The authors emphasize that, while such prescribing is often clinically unavoidable, it occurs in a context of limited high-quality evidence and heightened vulnerability, underscoring the ethical imperative for careful risk–benefit assessment, enhanced monitoring, and explicit communication with legal guardians (41).

The practice of off-label prescribing in pediatric psychiatry places professionals before a fundamental ethical imperative: the duty to treat, even in the absence of robust evidence of efficacy or safety for that specific population. International guidelines recognize this moral and legal obligation; however, such conduct must be accompanied by clear and detailed communication with patients’ legal guardians (42). In this context, informed consent should encompass not only the known risks and benefits of therapy but also the uncertainties arising from the lack of formal validation of drug use in the pediatric population (43).

From a clinical perspective, although many reports indicate satisfactory therapeutic outcomes, the risks associated with off-label practices should not be underestimated. Minghetti et al. (44) warn that, despite potential clinical benefits, the evidence base regarding safety and efficacy in children and adolescents remains limited. This situation is further compounded by the concomitant use of multiple psychotropic drugs—a practice known as polypharmacy—which affects up to 31.5% of pediatric psychiatric patients in certain samples (40). Such a scenario requires constant vigilance regarding drug interactions, adverse effects, and long-term impacts on neuropsychological development.

Beyond the individual implications for patients, off-label prescribing in children and adolescents raises structural questions about regulatory oversight and the responsibility of health systems to promote clinical research focused on this population (45). The lack of randomized controlled trials specifically designed for pediatric patients constitutes an ethical gap that limits prescribing precision and exposes patients to potentially avoidable risks. In this sense, the development of more specific clinical guidelines and the expansion of clinical trials that representatively include this age group are urgent needs (46).

Thus, the off-label prescribing of psychotropics in children and adolescents, although often unavoidable, should be conducted with prudence, grounded in the best available evidence, supported by detailed informed consent, and rigorously monitored (45). When appropriately guided, this practice can provide relevant therapeutic benefits but requires psychiatrists to exercise ethical sensitivity, technical expertise, and commitment to the safety of pediatric patients (43).

#### 4.4.2 Off-label practices in pregnant women

The prescription of psychotropic medications to pregnant women, particularly in an off-label context, represents one of the most complex scenarios in clinical psychiatry. This complexity arises from the delicate balance between the potential risks to the fetus and the indispensable therapeutic benefits for the mother’s mental health. Although off-label prescribing is common in this population, the scarcity of robust clinical data constitutes a critical gap that poses substantial ethical, legal, and scientific challenges (47).

Historically, pregnant women have been systematically excluded from clinical trials, primarily due to concerns regarding fetal safety. While this exclusion was motivated by precautionary principles, it has resulted in a limited evidence base to guide the safe prescription of medications during pregnancy (48). Consequently, psychiatrists often face the need to prescribe drugs outside of approved indications in a context of therapeutic uncertainty. Although the reviewed literature documents this therapeutic uncertainty, from a bioethical perspective, we argue that this reality contrasts with principles of autonomy and justice, which support the inclusion of pregnant women in clinical research, enabling them to benefit from therapeutic advances through informed decision-making (48).

Moreover, the interdependence between maternal and fetal health reinforces the importance of conducting ethical studies involving pregnant women. Failure to intervene, or undertreatment of mental disorders during pregnancy, can result in severe consequences for both mother and fetus, including maternal psychiatric decompensation and unfavorable obstetric outcomes (48, 49).

From a regulatory perspective, current legal frameworks often do not adequately address the needs of this specific population. The absence of clear guidelines and the rigidity of certain ethical and legal standards constitute significant barriers to conducting clinical research in pregnant women (47). Regulatory reforms and innovative frameworks are urgently needed to enable safe and ethical research with this population, simultaneously ensuring protection and equity (47, 49).

Initiatives such as the Drug Information Association (DIA) Medicines in Pregnancy Forum, held in 2014, highlighted the urgent need to promote the inclusion of pregnant women in clinical studies, provided that such research is conducted under carefully designed conditions and with strict ethical protection protocols (50). Systematic strategies for the collection and dissemination of data on drug use during pregnancy have also proven essential for supporting evidence-based clinical decision-making (50).

Thus, although the off-label use of psychotropics during pregnancy remains a frequently unavoidable practice, it is imperative to advance scientific knowledge production and regulatory reforms that ensure therapeutic safety and efficacy (47). Maintaining the status quo, characterized by exclusion and data omission, perpetuates an ethical and clinical paradox: protecting the fetus while denying the mother access to appropriate treatments may result in equally severe harm (51). Therefore, addressing these issues requires joint action among researchers, regulatory agencies, and bioethics bodies to promote safe, equitable, and evidence-based psychiatric practices for pregnant women (52).

Qualitative studies further indicate that psychiatrists often perceive off-label prescribing as ethically acceptable when supported by clinical experience, patient dialogue, and contextual vulnerability, even in the absence of robust trial data (17, 53).

### 4.5 limitations

This scoping review has some limitations. Although a systematic search was conducted across multiple databases and complemented by a clinical trial registry, database coverage restrictions and access limitations may have led to the omission of some relevant studies. The review may also be subject to language bias, as only studies published in English, Portuguese, and Spanish were included. In addition, as a scoping review, no formal assessment of methodological quality or risk of bias was performed; therefore, the findings should be interpreted as descriptive and exploratory rather than inferential. Finally, thematic mapping and keyword co-occurrence analyses depend on the consistency of author-provided keywords, which may vary across publications despite standardization efforts.

## 5 Final considerations

The keyword co-occurrence analysis revealed that the literature on off-label prescribing in psychiatry is characterized by a strong centrality of ethical, legal, and regulatory themes, particularly concerning informed consent and the protection of human rights. The observed clusters demonstrate that the field’s main concerns involve prescriber responsibility, patient safety risks, and the challenges posed by caring for vulnerable populations such as children, adolescents, and pregnant women. This thematic structure, reinforced by graph visualization, suggests that advances in clinical practices and in the regulation of off-label prescribing depend on the consolidation of interdisciplinary policies and the promotion of collaborative research capable of integrating ethical, clinical, and legal perspectives, thereby ensuring safer, more equitable psychiatric care aligned with patients’ fundamental rights.

The relationship between off-label prescribing and human rights, particularly the right to quality healthcare, is complex and multifaceted. While off-label prescribing may sometimes be necessary to ensure patient care, it raises significant ethical and legal issues related to informed consent and patient safety. This practice can affect the fundamental right to health, as patients may be exposed to unproven treatments without adequate information about potential risks.

In summary, off-label prescribing, when conducted without transparency, proper consent, and scientific support, may constitute a threat to human rights in healthcare. In this regard, public policies and clinical guidelines must prioritize the regulation of the practice, promote professional education on its ethical and legal implications, and ensure protective mechanisms that safeguard patients’ rights to safe, informed, and consensual treatments.

## Data Availability

The original contributions presented in the study are included in the article/**Supplementary Material**. Further inquiries can be directed to the corresponding author.
