# Supplementary material for: Thematic mapping of off-label prescription in psychiatry and its implications for bioethics, human rights, and clinical practice: a scoping review
Source: Front Psychiatry. 2026 Feb 13;17:1705340. doi: 10.3389/fpsyt.2026.1705340 (PMC12946064; doi:10.3389/fpsyt.2026.1705340)
Supplement: Supplementary file 1 [file DataSheet1.pdf]

Supplementary Material S1 — Full Electronic Search Strategies (Verbatim)

S1.1 PubMed (MEDLINE)

Database/platform: PubMed (National Library of Medicine)
Search fields: MeSH terms (controlled vocabulary)
Search query (verbatim):

(("Psychiatry"[Mesh]) AND "Off-Label Use"[Mesh]) AND ("Off-Label Use/economics"[Mesh] OR "Off-Label Use/ethics"[Mesh] OR "Off-Label Use/history"[Mesh] OR "Off-Label Use/legislation and jurisprudence"[Mesh] OR "Off-Label Use/standards"[Mesh] OR "Off-Label Use/statistics and numerical data"[Mesh])

Limits/filters applied:

- Publication date: July 2024 to July 2025 (search execution window).
- Eligibility screening restriction (applied during screening stage, not as a database filter): last 15 years, with exceptions for historically foundational studies.

Search execution notes:

- The query combines *Psychiatry* and *Off-Label Use* MeSH headings and expands the scope by explicitly including key MeSH subheadings relevant to public mental health and normative dimensions (economics, ethics, history, legislation and jurisprudence, standards, statistics and numerical data).
- All retrieved records were exported to the reference manager (Mendeley Reference Manager) for deduplication and screening.

S1.2 Virtual Health Library (VHL/BVS)

Database/platform: Biblioteca Virtual em Saúde (BVS/VHL)
Search fields: free-text terms (Portuguese) entered in the BVS search interface
Search query (verbatim):

(psiquiatria) AND (Uso off-label) OR (ética) OR (história) OR (educação) OR (legislação & jurisprudência) OR (organização & administração) OR (normas) OR (estatística & dados numéricos) OR (tendências)

Limits/filters applied:

- Search execution window: July 2024 to July 2025.
- Eligibility screening restriction (applied during screening stage, not as a database filter): last 15 years, with exceptions for historically foundational studies.

Search execution notes:

- The search strategy was designed to capture publications addressing off-label prescribing in psychiatry and related normative domains (ethics, history, legislation/jurisprudence, standards, administration/organization, and numerical/statistical data), including literature relevant to Latin America and Portuguese-language contexts.
- All retrieved records were exported and consolidated in the reference manager (Mendeley Reference Manager) for deduplication and screening.

Important note on Boolean logic (for reproducibility)

Because the BVS interface may interpret operator precedence differently depending on configuration, we preserved the query exactly as executed. For transparency and to support replication, we also recommend reporting the logically grouped form used to operationalize the intent of the query during search refinement (if applicable in your interface), ensuring that all OR-terms are nested within parentheses and combined with the AND-terms.

S1.2.a Virtual Health Library (VHL/BVS) — Normalized Logic

(psiquiatria) AND

(

("uso off-label") OR

(ética) OR

(história) OR

(educação) OR

("legislação & jurisprudência") OR

("organização & administração") OR

(normas) OR

("estatística & dados numéricos") OR

(tendências)

)

Operational notes:

- The normalized logic nests all OR-terms within a single parenthetical block combined with the AND-term (*psiquiatria*), ensuring that retrieved records explicitly relate to psychiatry while capturing normative, ethical, historical, legal, organizational, and quantitative dimensions of off-label use.

- This normalized representation reflects the intended conceptual scope applied during screening and does not alter the original verbatim query reported above.

S1.3 SciSpace

SciSpace (https://scispace.com) was used as a **complementary discovery and mapping tool**, not as a primary bibliographic database.

Search approach (verbatim):

- Query terms:
  - "Off-label prescribing in psychiatry"
  - "Off-label prescription AND human rights"
  - "Informed consent AND off-label psychiatry"
  - "Off-label psychotropic drugs AND ethics"
  - "Off-label prescribing AND pregnant women"
  - "Off-label prescribing AND children AND psychiatry"

Operational use:

- Searches were performed across titles and abstracts indexed by SciSpace.
- Filters were applied for publication year (2010–2025) and peer-reviewed sources when available.
- SciSpace was used **exclusively** to identify potentially relevant articles and explore thematic proximity between concepts.

Methodological safeguards:

- No automatic content generation, data extraction, or analytical interpretation was performed by SciSpace.
- All records identified through SciSpace were independently screened by the authors according to predefined inclusion and exclusion criteria.
- Final inclusion decisions were based solely on manual full-text assessment.

S1.4 PsycINFO (American Psychological Association)

Database/platform: PsycINFO (APA)
Search fields: Any Field

Search query:

"Off-Label Use" AND "Psychiatry"

Filters applied:

- Publication years: 2020–2025

Search results and notes:

- PsycArticles: 0 records
- PsycINFO records: 197

Titles and abstracts were screened. The retrieved records were either duplicates of studies indexed in PubMed or other biomedical databases, or publications that did not address off-label prescribing in psychiatry as a primary ethical, legal, or clinical practice. PsycINFO is a subscription-based database with restricted full-text access. No eligible PsycArticles were identified.

S1.5 ClinicalTrials.gov

Registry/platform: ClinicalTrials.gov
Condition/Disease: Psychiatry
Other terms: Off Label-use

Search results and notes:

- One registered study identified: **NCT01623505**

The identified trial focused on smoking cessation pharmacotherapy and cardiovascular outcomes, with psychiatric status assessed only as a secondary stratification variable. As off-label prescribing in psychiatry was not the primary focus of the study, it did not meet the predefined eligibility criteria and was excluded.

S1.6 Web of Science (Core Collection)

Database: Web of Science Core Collection
Search date: Dezember 2025

Search strategy (verbatim):

"Off-Label Use" AND "Psychiatry"

Filters applied:

- Document types: Article, Review
- Research areas: Psychiatry, Behavioral Sciences, Ethics, Health Policy
- Languages: English

Results retrieved: 8 records

Screening outcome:

- Duplicates removed: 3
- Excluded due to lack of full-text access: 1
- **Included studies:** 4

These four studies were incorporated into the qualitative synthesis and discussion.

S1.7 Embase

Database: Embase

Access status:
Full access to Embase was **not available** to the research team at the time of the review due to institutional and subscription limitations.

Action taken:
The database was documented as a planned source; however, no search could be executed. This limitation is explicitly acknowledged in the manuscript.

S1.8 Scopus

Database: Scopus

Access status:
Full access to Scopus was **not available** to the research team at the time of the review due to institutional and subscription limitations.

Action taken:
As with Embase, Scopus is reported transparently as an inaccessible database, and this is discussed as a methodological limitation.

Summary of Database Coverage

Source	Records Retrieved
PubMed	21
BVS	1,748
SciSpace	50

Source	Records Retrieved
Web of Science	8
PsycINFO (PsycArticles)	0
ClinicalTrials.gov	1
Embase	Not accessible
Scopus	Not accessible

Summary Note

All databases and registries consulted are reported to ensure transparency and reproducibility, in accordance with PRISMA-ScR recommendations. Searches were conducted in PubMed, the Virtual Health Library (BVS), Web of Science, PsycINFO (APA), and ClinicalTrials.gov. Searches in PsycINFO and ClinicalTrials.gov retrieved records; however, none met the eligibility criteria for addressing off-label prescribing in psychiatry as a primary ethical, legal, or clinical practice focus.

The search in Web of Science identified eight records, of which four were excluded due to duplication or lack of access to full text, resulting in four eligible studies that were included in the synthesis. Searches in Embase and Scopus could not be fully executed due to institutional access restrictions; this limitation is explicitly acknowledged. Overall, the search results reinforce that the available evidence on off-label prescribing in psychiatry is predominantly framed within normative, regulatory, and public health perspectives rather than empirical clinical trials.
