# Supplementary material for: Thematic mapping of off-label prescription in psychiatry and its implications for bioethics, human rights, and clinical practice: a scoping review
Source: Front Psychiatry. 2026 Feb 13;17:1705340. doi: 10.3389/fpsyt.2026.1705340 (PMC12946064; doi:10.3389/fpsyt.2026.1705340)
Supplement: Supplementary file 2 [file DataSheet2.pdf]

International Platform of Registered Systematic Review and Meta-analysis Protocols

INPLASY

Thematic Mapping of Off-Label Prescription in Psychiatry and Its Implications for Bioethics, Human Rights, and Clinical Practice: A Scoping Review

INPLASY202590058

doi: 10.37766/inplasy2025.9.0058

Received: 14 September 2025

Published: 14 September 2025

dos Santos, HMM; Botelho-Souza, LF; Nunes, R; Duarte,I.

Corresponding author:
Luan Felipo Botelho-Souza

luan_botelho@hotmail.com

Author Affiliation:
CENTRO UNIVERSITÁRIO APARÍCIO CARVALHO FIMCA.

ADMINISTRATIVE INFORMATION

Support - OWN SUPPORT.

Review Stage at time of this submission - Completed but not published.

Conflicts of interest - None declared.

INPLASY registration number: INPLASY202590058

Amendments - This protocol was registered with the International Platform of Registered Systematic Review and Meta-Analysis Protocols (INPLASY) on 14 September 2025 and was last updated on 14 September 2025.

INTRODUCTION

Review question / Objective Central Question What are the ethical, legal, and clinical implications of off-label prescribing in psychiatry, with emphasis on informed consent and the protection of human rights?
Objective
To map and analyze the scientific literature on off-label prescribing in psychiatry, identifying its impacts on bioethical principles, human rights, and clinical practice.

Background Mental disorders represent a significant challenge to global public health. According to the World Health Organization (WHO), approximately one in eight people worldwide lives with a mental disorder, with depression, anxiety disorders, and substance use disorders standing out as the most prevalent (WHO, 2022).
In the field of psychiatry, clinical practice often requires complex therapeutic decisions, particularly in the face of the scarcity of approved treatments for certain clinical conditions, age groups, or vulnerable populations. In such circumstances, it is common to adopt off-label prescribing, which is characterized by the use of medications outside the indications officially approved by regulatory agencies, whether regarding indication, dosage, route of administration, or age group (Radley; Finkelstein; Stafford, 2006).
Although legal and, in many cases, supported by emerging scientific evidence, off-label practices may generate uncertainties regarding treatment efficacy and safety, raising important ethical and legal dilemmas. In this context, the fundamental bioethical principles of autonomy, beneficence, non-maleficence, and justice become essential to guide responsible clinical conduct (Beauchamp; Childress, 2019). Moreover, it must be recognized that access to safe, effective, and high-quality care constitutes a fundamental human right, as established by various international declarations and WHO guidelines (ONU, 1948; WHO, 2021). The decision to prescribe off-label medications

dos Santos et al. INPLASY protocol 202590058. doi:10.37766/inplasy2025.9.0058 Downloaded from https://inplasy.com/inplasy-2025-9-0058/

therefore requires careful analysis that considers not only the available scientific evidence and bioethical values but also the commitment to human rights, particularly respect for patient dignity, safety, and well-being.

Rationale Off-label prescribing in psychiatry is a widely disseminated clinical practice, particularly given the limited number of approved treatments for certain conditions and specific populations such as children, adolescents, pregnant women, and the elderly. Despite its clinical relevance, this practice remains surrounded by uncertainties regarding treatment efficacy and safety, while also raising complex ethical, legal, and regulatory dilemmas.

The international debate on this topic has underscored the need to deepen the understanding of the intersections between bioethics, human rights, and clinical practice, considering that the absence of clear consensus may compromise both patient safety and professional autonomy. In this context, conducting a Scoping Review is pertinent, as it allows for the systematic mapping of existing scientific production, identification of knowledge gaps, analysis of thematic trends, and provision of evidence to support the development of public policies, clinical guidelines, and future research.

Therefore, this study is justified by the urgent need to consolidate evidence that supports psychiatric practices which are safer, more equitable, and aligned with bioethical principles and human rights, thereby contributing to the improvement of mental health care at both national and international levels.

METHODS

Strategy of data synthesis Data synthesis was conducted through a narrative and thematic approach, based on the standardized extraction of essential information from the selected studies (authors, year, setting, objectives, methodology, results, and implications). Controlled descriptors from MeSH (Medical Subject Headings) were applied, including "Off-Label Use" and "Psychiatry", along with related terms such as "Informed Consent", "Human Rights", and "Ethics".

Searches were performed in the electronic databases PubMed and the Virtual Health Library (VHL), complemented by the SciSpace platform as a discovery and mapping tool for scientific literature. In addition, sources of grey literature were incorporated through Google Scholar, institutional documents from the World Health Organization (WHO), and national and international regulatory agencies.

The synthesis integrated both narrative analysis and keyword co-occurrence analysis, employing an incidence matrix and visualizations such as heatmaps and co-occurrence graphs using VOSviewer software, in order to identify thematic patterns, knowledge gaps, and conceptual interrelationships.

Eligibility criteria Studies published within the last 15 years in Portuguese, English, or Spanish that addressed off-label prescribing in psychiatry and discussed its bioethical, clinical, or legal implications were included. Exceptions were made for historical studies considered fundamental to the field.

Exclusion criteria comprised articles that did not specifically address psychiatry, did not mention bioethical principles, or provided insufficient data for analysis.
In addition, grey literature sources (technical reports, institutional guidelines, and documents from regulatory agencies) were considered when they met the criteria of relevance and scientific rigor.

Source of evidence screening and selection The selection process was conducted in three sequential stages:
1. Initial screening of titles and abstracts, independently performed by two reviewers to exclude clearly irrelevant studies;
2. Full-text assessment, applied to potentially eligible studies, based on the predefined inclusion and exclusion criteria;
3. Data extraction and validation, using a standardized form.

Discrepancies between reviewers at any stage were resolved through consensus discussions; when consensus was not achieved, a third reviewer was consulted for final decision-making.
The entire screening and selection process was documented in a PRISMA-ScR flow diagram, ensuring transparency, traceability, and methodological rigor.

Data management Data management was performed using Mendeley Reference Manager to organize references, remove duplicates, and facilitate collaborative screening. All citations imported from the databases (PubMed, VHL, and SciSpace) were integrated into a single reference library.

dos Santos et al. INPLASY protocol 202590058. doi:10.37766/inplasy2025.9.0058 Downloaded from https://inplasy.com/inplasy-2025-9-0058/

During screening, each study was classified according to the predefined inclusion and exclusion criteria, and decisions were recorded directly in the reference manager. For data extraction, a standardized form was applied, covering information on authorship, year, journal, country, objectives, methodology, main results, and bioethical and clinical implications.

Data consistency was verified by two independent reviewers, and any discrepancies were resolved through consensus or, when necessary, by consulting a third reviewer.

Reporting results / Analysis of the evidence The reporting of results followed the PRISMA-ScR recommendations, ensuring transparency and reproducibility. The included studies were synthesized in evidence tables and matrices, covering variables such as authorship, year, country, study type, population, objectives, methodology, and main findings.

Evidence analysis was conducted in two complementary stages:
1. Descriptive and narrative analysis, organized by thematic categories (bioethics, human rights, regulation, informed consent, and special populations), highlighting convergences, divergences, and knowledge gaps;
2. Keyword co-occurrence analysis, performed through heatmaps and co-occurrence graphs using VOSviewer, in order to identify conceptual clusters, thematic patterns, and interrelationships among the most recurrent topics.

This integrated approach enabled characterization of the current state of scientific production, identification of research trends, and indication of priority areas for future investigations.

Presentation of the results The analysis of results was conducted in a descriptive, thematic, and exploratory manner, consistent with the objectives of the Scoping Review. Initially, a narrative synthesis of the studies was performed, categorizing the evidence into the following main axes: bioethics, human rights, regulation, informed consent, and special populations.

Complementarily, a keyword co-occurrence analysis was applied using VOSviewer software, which enabled the construction of heatmaps and co-occurrence graphs. This strategy allowed the identification of conceptual clusters, central thematic cores, and peripheral subthemes.

The presentation of results was structured in different formats:
1. Tables and evidence matrices, highlighting methodological characteristics, objectives, and main findings of each study;
2. Graphical visualizations (heatmaps and graphs), illustrating connection patterns between descriptors and topics;
3. Integrated narrative discussion, linking thematic findings to bioethical, clinical, and regulatory implications.

This process ensured a comprehensive and systematized view of the body of evidence, providing greater clarity in identifying gaps, trends, and practical implications.

Language restriction English, Portuguese and Spanish.

Country(ies) involved Brazil and Portugal.

Keywords Off-Label Use; Psychiatry; Bioethics; Human Rights; Informed Consent.

Contributions of each author
Author 1 - Humberto Müller Martins dos Santos - Conception, data search and selection, writing, and discussion.
Email: contato@humbertomuller.com.br
Author 2 - Luan Felipo Botelho-Souza - Data search execution, data selection, writing, data analysis, and results presentation.
Email: luan_botelho@hotmail.com
Author 3 - Rui Nunes - Supervision and review.
Email: ruinunes@med.up.pt
Author 4 - Ivone Duarte - Overall supervision, data selection, and review.
Email: iduarte@med.up.pt

dos Santos et al. INPLASY protocol 202590058. doi:10.37766/inplasy2025.9.0058 Downloaded from https://inplasy.com/inplasy-2025-9-0058/
