# Supplementary material for: Thematic mapping of off-label prescription in psychiatry and its implications for bioethics, human rights, and clinical practice: a scoping review
Source: Front Psychiatry. 2026 Feb 13;17:1705340. doi: 10.3389/fpsyt.2026.1705340 (PMC12946064; doi:10.3389/fpsyt.2026.1705340)
Supplement: Supplementary file 3 [file DataSheet3.pdf]

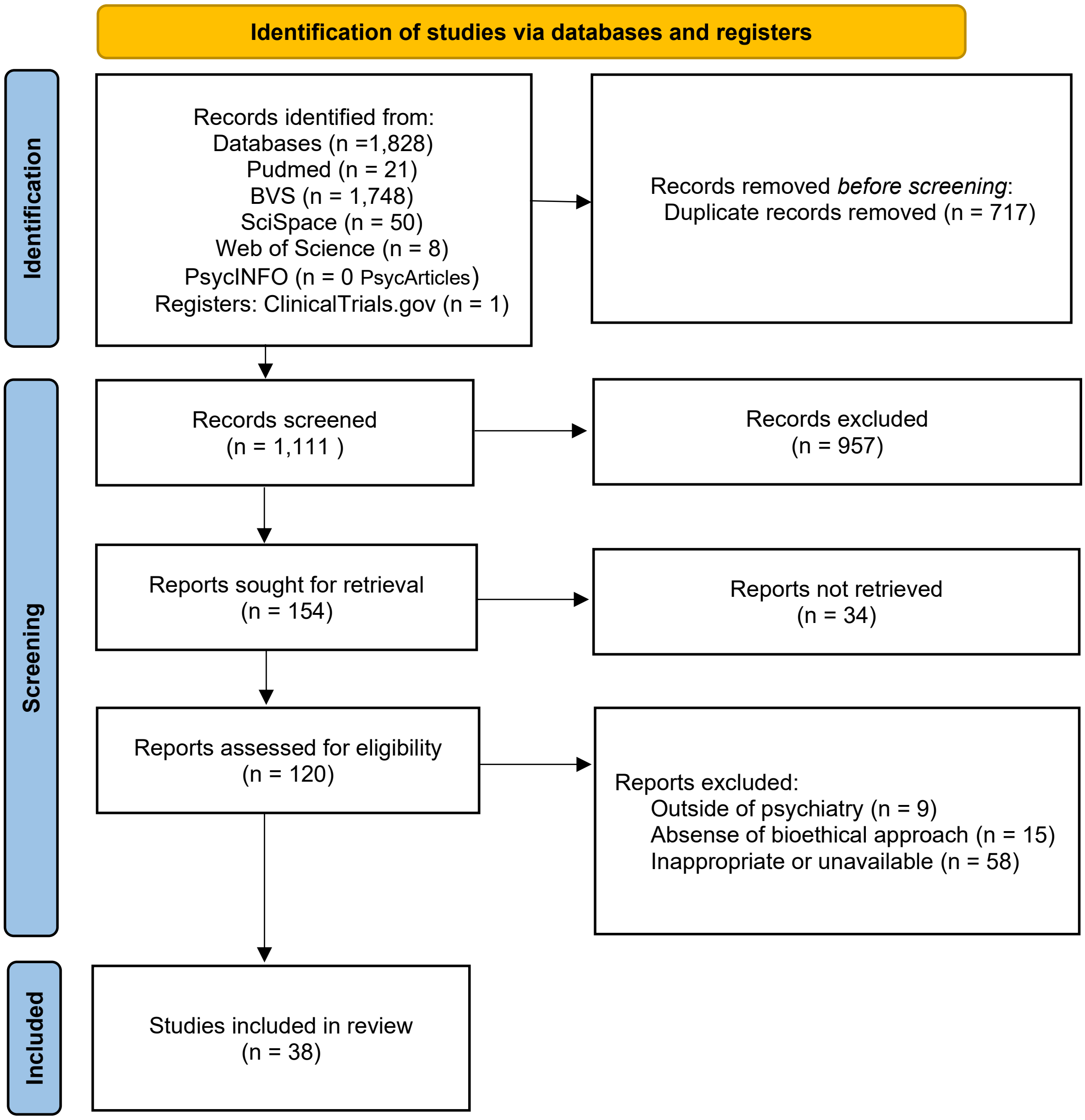

Source: Page MJ, et al. BMJ 2021;372:n71. doi: 10.1136/bmj.n71.

This work is licensed under CC BY 4.0. To view a copy of this license, visit https://creativecommons.org/licenses/by/4.0/
