# Supplementary material for: Thematic mapping of off-label prescription in psychiatry and its implications for bioethics, human rights, and clinical practice: a scoping review
Source: Front Psychiatry. 2026 Feb 13;17:1705340. doi: 10.3389/fpsyt.2026.1705340 (PMC12946064; doi:10.3389/fpsyt.2026.1705340)
Supplement: Supplementary file 4 [file Table1.docx]

# Table 1. Evidence Matrix of Selected Studies

| **Author (Year)** | **Abbreviated Title** | **Publication Venue / Journal** | **Year** | **Country/Region** | **Publication Type** | **Literature Type** | **Category** | **Abstract / Main Findings** | **Themes** | **Keywords** |
| --- | --- | --- | --- | --- | --- | --- | --- | --- | --- | --- |
| AVRAM et al. (2022) | Annotating off-label drug usage | medRxiv | 2022 | International | Preprint | Grey | Human Rights / Public Health | Discusses annotation and data mining of off-label use from alternative and unconventional sources. | Off-label use; Unconventional sources; Data mining | off-label, pharmacovigilance, grey literature, data mining, medicines |
| BLEASE et al. (2018) | Informed consent in psychotherapy | Journal of Contemporary Psychotherapy | 2018 | USA/UK | Review | White | Informed Consent | Debates the need for informed consent based on evidence and individual factors. | Informed consent; Psychotherapy; Evidence-based practice | informed consent, psychotherapy, ethics, disclosure, evidence-based practice |
| BLONDON et al. (2008) | [Off-label prescribing] | Revue médicale suisse | 2008 | Switzerland | Review | White | Legal Risks / Litigation | Analyzes ethical-legal challenges, risks, and practices in off-label prescribing. | Off-label prescribing; Regulatory issues; Medical ethics | off-label, psychiatry, regulation, informed consent, medical liability |
| BRAÜNER et al. (2016) | Off-Label Prescription in Child Psychiatry | Journal of Clinical Psychopharmacology | 2016 | Denmark | Original article | White | Pediatric Population | High prevalence of off-label prescribing in children/adolescents; highlights risks and need for monitoring. | Off-label in child psychiatry; Polypharmacy; Safety | off-label, child psychiatry, psychotropics, polypharmacy, risks, prevalence |
| CHISOLM & PAYNE (2016) | Psychotropic drugs in pregnancy | BMJ (Online) | 2016 | UK | Review | White | Pregnant Population | Reviews psychotropic drug management during pregnancy; highlights gaps in evidence. | Psychotropics in pregnancy; Clinical management; Fetal risk | pregnancy, psychotropics, safety, off-label, clinical management, fetal risk |
| CLEMOW et al. (2015) | Medicines in Pregnancy Forum Proceedings | Therapeutic Innovation & Regulatory Science | 2015 | International | Forum report | Grey | Pregnant Population / Regulation | Highlights the need for innovative regulatory and ethical strategies in research with pregnant women. | Ethics in pregnancy medication; Regulation; Clinical research | ethics, pregnancy, medicines, regulation, clinical research |
| COLEMAN & ROSOFF (2011) | Legal Regulation of Physicians’ Off-Label Prescribing | Notre Dame Law Review | 2011 | USA | Original article | White | Human Rights / Ethics / Regulation | Argues for legal regulation of off-label prescribing and its impact on patient autonomy. | Legal regulation; Off-label prescribing; Right to health | regulation, off-label, legislation, medical liability, right to health |
| COMANOR & NEEDLEMAN (2016) | Law, Economics, and Medicine of Off-Label Prescribing | Washington Law Review | 2016 | USA | Original article | White | Human Rights / Economics / Health | Examines economic and legal impacts of off-label prescribing on public health and the pharmaceutical market. | Health economics; Off-label; Drug policy | economics, off-label, regulation, drug policy, legal analysis |
| CONKO (2010) | Truth or Consequences: Perils and Protection | Social Science Research Network | 2010 | USA | Article / Working paper | Grey | Litigation / Public Policy | Analyzes legal consequences of promoting and disseminating off-label use. | Off-label promotion; Freedom of speech; Regulation | off-label, promotion, advertising, FDA, freedom of speech |
| COPPOLA et al. (2024) | Medicines in pregnancy: extrapolation framework | Pharmacometrics and Systems Pharmacology | 2024 | International | Review | White | Pregnant Population / Pharmacology | Proposes methods for extrapolating pharmacology data to pregnant women. | Pharmacology in pregnancy; Clinical extrapolation; Evidence gaps | pregnancy, clinical pharmacology, extrapolation, knowledge, safety |
